# Are freestanding midwifery units a safe alternative to obstetric units for low-risk, primiparous childbirth? An analysis of effect differences by parity in a matched cohort study

**DOI:** 10.1186/s12884-016-1208-1

**Published:** 2017-01-09

**Authors:** Louise Fischer Christensen, Charlotte Overgaard

**Affiliations:** 1 Department of Health Science and Technology, Faculty of Medicine, Aalborg University, Aalborg, Denmark; 2 Department of Gynecology & Obstetrics, Aalborg University Hospital, Sdr. Skovvej 15, DK-9000 Aalborg, Denmark

**Keywords:** Freestanding midwifery unit, Birth centre, Primiparity, Low risk women, Parity, Childbirth, Birth Outcomes

## Abstract

**Background:**

Intrapartum complications and the use of obstetric interventions are more common in primiparous childbirth than in multiparous childbirth, leading to concern about out of hospital birth for primiparous women. The purpose of this study was to determine whether the effect of birthplace on perinatal and maternal morbidity and the use of obstetric interventions differed by parity among low-risk women intending to give birth in a freestanding midwifery unit or in an obstetric unit in the North Denmark Region.

**Methods:**

The study is a secondary analysis of data from a matched cohort study including 839 low-risk women intending birth in a freestanding midwifery unit (primary participants) and 839 low-risk women intending birth in an obstetric unit (individually matched control group). Analysis was by intention-to-treat. Conditional logistic regression analysis was applied to compute odds ratios and effect ratios with 95% confidence intervals for matched pairs stratified by parity.

**Results:**

On no outcome did the effect of birthplace differ significantly between primiparous and multiparous women. Compared with their counterparts intending birth in an obstetric unit, both primiparous and multiparous women intending birth in a freestanding midwifery unit were significantly more likely to have an uncomplicated, spontaneous birth with good outcomes for mother and infant and less likely to require caesarean section, instrumental delivery, augmented labour or epidural analgesia (although for caesarean section this trend did not attain statistical significance for multiparous women). Perinatal outcomes were comparable between the two birth settings irrespective of parity. Compared to multiparas, transfer rates were substantially higher for primiparas, but fell over time while rates for multiparas remained stable.

**Conclusions:**

Freestanding midwifery units appear to confer significant advantages over obstetric units to both primiparous and multiparous mothers, while their infants are equally safe in both settings. Our findings thus support the provision of care in freestanding midwifery units as an alternative to care in obstetric units for all low-risk women regardless of parity. In view of the global rise in caesarean section rates, we consider it an important finding that freestanding midwifery units show potential for reducing first-birth caesarean.

**Electronic supplementary material:**

The online version of this article (doi:10.1186/s12884-016-1208-1) contains supplementary material, which is available to authorized users.

## Background

It is well established that primiparous childbirth differs in many respects from multiparous childbirth. Compared to multiparous women, primiparas have longer labours [1–3], are at an increased risk of intrapartum complications [4–7] and undergo substantially more obstetric interventions [8–11]. The use of interventions, especially assisted vaginal delivery and unplanned caesarean delivery, has been found to have a negative impact on women’s birth experience, which may partly explain why primiparous birth experiences are more negative [12–14].

Recent decades have seen a major rise in overall caesarean section rates worldwide [15–17]. In Denmark, the caesarean section rate has thus increased from 13.1% in 1997 to 22.4% in 2013 and have since stabilised at this level [18]. Several newer observational studies provide evidence that the greatest contributor to this development is a sharp increase in caesarean delivery among low-risk primiparous women, leading to a subsequent increase in repeat caesarean sections [19–27]. This trend should be seen in light of the progressive increase in hospitalised childbirths witnessed in most high- and middle-income countries over the last century. Thus, the vast majority of women today give birth in increasingly centralised and specialised obstetric units (OUs) [28, 29]. The overriding reason for this is a concern with safety. Hospitalisation and the centralisation of childbirth, however, have coincided with a steady increase in obstetric intervention rates that have far exceeded clinically indicated levels [28–30], suggesting that OUs may not always provide an optimal setting for uncomplicated deliveries.

To counterbalance the predominantly technological and medical approach in OU settings, alternative birth settings such as freestanding midwifery units (FMUs) have been introduced, providing women with a choice of birthplace. Overall, FMUs offer individualised, low-technological care encouraging spontaneous, vaginal birth without routine intervention to low-risk women, often in a family-friendly environment close to home [31]. Specialist care is typically not readily available and requires transfer by ambulance to an OU. This, in particular, has given rise to a debate over safety [32–35]. As acute obstetric and/or neonatal complications cannot be excluded, even with careful risk assessment, concern has been voiced that untimely transfers may cause a critical delay in the access to specialised, obstetric care [32, 36]. However, there is increasingly strong evidence that care in an FMU can offer benefits for women with low-risk pregnancies and is safe for both the woman and infant [8, 37–43].

In the Danish birth centre study of care in FMUs versus OUs, we have previously found that low-risk women intending birth in an FMU had significantly lower morbidity and were significantly less likely to require obstetric intervention compared with low-risk women intending birth in an OU. Moreover, no significant differences in perinatal outcomes were found [37]. These findings have been confirmed by the landmark Birthplace in England research programme [8]. Yet, our systematic search of the literature identified only two studies that have investigated whether these effects do also apply to primiparous women [8, 44]. The available evidence suggests that, regardless of parity, care in an FMU is safe for the infant and offers benefits for the mother.

Due to the high rate of obstetric complications and interventions among primiparas, as compared to multiparas [4–11], care in FMUs for this group of women is particularly controversial. Safety concerns have been expressed, especially over the considerably higher transfer rates from FMUs to specialised obstetric units for primiparous women [36, 37, 45, 46]. One study in particular reported a higher incidence of urgent transfers among primiparas [36]. The suitability of care in FMUs for this group of women can thus be questioned and, accordingly, not all FMUs accept primiparous women [47]. Further evidence is therefore needed.

This study is based on data from the Danish birth centre study comparing FMU and OU settings for birth [37]. Our aim was to determine whether the effect of birthplace on perinatal and maternal morbidity, birth interventions, use of pain relief and birth positions differed by parity among low-risk women intending to give birth in an OU or FMU in the North Denmark Region. Given the reported large differences in transfer rates between primiparas and multiparas, we moreover aimed to describe transfer patterns for the two groups.

### Hypotheses

Based on the literature, we hypothesised that the effect of birthplace on perinatal and maternal morbidity, use of obstetric interventions and the likelihood of a spontaneous, uncomplicated birth would not differ by parity.

## Methods

A cohort study with a matched control group was conducted between March 2004 and October 2008, with consecutive sampling of data on 839 women intending birth in an FMU and a matched control group of 839 women intending birth in an OU. A detailed account of the study methodology is published elsewhere [37].

As the primary outcome for this analysis of effect modification by parity, we chose the composite outcome “a spontaneous, uncomplicated birth leaving both mother and infant in good condition”, which was developed and used in another substudy [48]. The outcome was defined by the following criteria: spontaneous onset of labour at 37 through 42 gestational weeks leading to spontaneous birth of an infant with a minimum Apgar score of 9 at 5 min combined with the absence of caesarean section, instrumental delivery, medical augmentation of labour, episiotomy, shoulder dystocia, third/fourth-degree perineal tearing, uterine rupture, retained placenta and postpartum bleeding >500 ml. Given that a women undergoing caesarean section in her first birth has a higher risk of complications, caesarean section and adverse outcomes in subsequent births [19, 49–54], caesarean section was chosen as a further primary outcome measure.

Secondary outcomes included admission to a neonatal intensive care unit (NICU) within the first 24 h postpartum, infant readmission 0–28 days postpartum, Apgar score of <9 at 5 min, intact perineum, third/fourth-degree tearing, maternal readmission, instrumental delivery, augmentation of labour, epidural analgesia, water birth and upright birth position.

### Setting

The overall study cohort was derived from data from two freestanding midwifery units and two obstetric units located in the North Denmark Region. In both types of setting, care for low-risk women was provided by midwives and all units followed the same multidisciplinary guidelines for referral and transfer.

All specialist obstetric services in the region were located at the two participating OUs, one of which was a highly specialised unit with approximately 3500 births a year, the other was in a regional hospital with approximately 1400 yearly births.

The two FMUs were located adjacent to community hospitals without on-site obstetric services, although, contrary to what is the case in some FMUs, intensive care and anaesthesiology services were available in case of emergencies. The average numbers of births in the FMUs were 170 and 130 annually. In case of complications, indication hereof or need for pharmacological pain relief, the woman and/or infant were transferred to the nearest OU/NICU. Transfers to OUs were by ambulance (although in non-urgent cases often in the family’s own car) with minimum transfer times of 25 and 35 min. To ensure the safety of women and infants, midwives in the FMUs were required to have completed obstetric emergency training and have at least 2 years of relevant work experience.

Care in the participating FMUs was characterised by one-to-one care and continuous support throughout labour, while this was usually not available until late in the first stage of labour in the participating OUs. Furthermore, midwives in the FMUs provided antenatal and intrapartum care in a team care model that increased the possibility that the women during birth would be cared for by a midwife they were familiar with.

Unit characteristics and differences in care concepts are more fully explained elsewhere [37].

### Participants

The overall study sample comprised 1678 women with low-risk pregnancies, intending birth in one of the participating FMUs (primary participants, *n* = 839) or OUs (matched controls, *n* = 839) during the 3.5-year study period.

All women who opted for birth in the FMUs were admitted on the basis of the rigorous criteria set out in regional guidelines according to which the women were considered to be at low obstetric risk if they presented with spontaneous onset of labour between 37 + 0 and 41 + 6 weeks of gestation following an uncomplicated pregnancy, with no condition to increase the risk of obstetric complications. A similar definition of low-risk criteria was later outlined in the National Institute for Health and Care Excellence (NICE) intrapartum care guidelines [55].

Each woman in the FMU group was matched with a woman intending to give birth in the nearest OU, thus forming the control group. The matching criteria were: low-risk status, parity, body mass index (BMI), age, smoking status, ethnicity, cohabitation status, education level and occupation level. Women intending OU birth were included in the control group only if they represented a strict match on all nine criteria at the start of care in labour (details presented in Table 1). The matching yielded two fully comparable groups, as exhaustively described in [37].

**Table 1:** Distribution of matching characteristics by birthplace

Characteristics	FMU (*n* = 839)		OU (*n* = 839)	
	*N*	(%)	*N*	(%)
Risk status				
Low obstetric risk	839	(100)	839	(100)
Parity				
Primiparous women	215	(25.6)	215	(25.6)
Multiparous women	624	(74.4)	624	(74.4)
Smoking status				
Non-smokers	684	(81.5)	684	(81.5)
Smokers	155	(18.5)	155	(18.5)
Ethnicity				
Nordic or Western European	805	(96)	809	(96.4)
Other ethnicity	34	(4.0)	30	(3.6)
Cohabitation status				
Living with partner	815	(97.1)	819	(97.6)
Living alone	24	(2.9)	20	(2.4)
Education level				
No postsecondary education	230	(27.4)	230	(27.4)
Postsecondary education	609	(72.6)	609	(72.6)
Occupation level				
Low level of employment^a^	535	(63.8)	535	(63.8)
High level of employment	304	(36.2)	304	(36.2)
	Mean (SD)		Mean (SD)	
Body Mass Index (BMI)^b^	24.2	(3.9)	24.0	(3.9)
Age^b^	29.4	(4.6)	30.2	(4.5)

^a^Unskilled work, vocational work or work requiring 1–2 years of postsecondary education

^b^Matched within a range of +/- 5

### Data collection

In each of the participating units one or two midwives acted as project staff. Following our written instructions, data on sociodemographic characteristics, present and previous pregnancies and births, neonatal outcomes and transfers were collected.

Approval for this study was obtained from the Danish Data Protection Agency, (reference number: 2005-41-5352). Ethical approval is not needed for this type of study. The collection and management of data were carried out in strict accordance with Danish legislation on personal data processing [56].

### Statistical analysis

For this secondary data analysis, the two overall groups were dichotomised by parity for subgroup analysis to test for effect modifications. While no separate power calculations were performed for this study, the power calculations that were carried out as part of the overall study are accounted for in [37].

For all outcome measures, odds ratios and effect ratios with 95% confidence intervals were determined for matched pairs (overall and stratified by parity) by conditional logistic regression analysis. Calculated p-values were two-sided and considered statistically significant when below 0.05. All data were analysed in accordance with the intention-to-treat principle, using STATA software version 11.

## Results

Of the 1678 low-risk women in our data set, 430 (25.6%) were primiparous, while 1248 (74.4%) were multiparous. None were lost to follow-up (see Additional file 1: study flow chart). Table 2 presents the results of conditional logistic regression analysis of the parity-induced subgroups and effect differences.

**Table 2:** Effect of birthplace on birth outcomes by parity

	Primiparous women FMU/OU (*n* = 215)/(*n* = 215)	Multiparous women FMU/OU (*n* = 624)/(*n* = 624)	Effect ratio Primiparas/multiparas
	OR (95% CI)	OR (95% CI)	OR (95% CI)
Primary outcomes			
Uncomplicated birth	2.2 (1.4–3.3)	2.9 (2.0–4.2)	0.7 (0.4–1.3)
Caesarean section	0.4 (0.2–0.9)	0.8 (0.3–2.2)	0.6 (0.2–2.1)
Secondary outcomes			
Perinatal:			
Apgar score <9/5 min	0.6 (0.2–1.9)	0.8 (0.4–1.9)	0.8 (0.2–3.0)
NICU admission <24 h	0.8 (0.4–1.9)	0.7 (0.3–1.8)	1.2 (0.3–4.3)
Infant readmission	0.4 (0.1–1.4)	0.7 (0.4–1.3)	0.6 (0.2–2.2)
Maternal:			
Intact perineum	1.3 (0.9–1.9)	1.3 (1.02–1.7)	1.0 (0.6–1.6)
3^rd^-4^th^ degree tear	0.9 (0.4–2.0)	0.6 (0.2–1.7)	1.7 (0.4–6.4)
Maternal readmission	0.3 (0.1–0.9)	0.8 (0.4–1.4)	0.4 (0.1–1.3)
Other:			
Instrumental delivery	0.4 (0.2–0.7)	0.3 (0.1–0.9)	1.5 (0.4–6.2)
Augmentation of labour	0.4 (0.3–0.6)	0.3 (0.2–0.5)	1.3 (0.6–2.7)
Epidural analgesia	0.4 (0.3–0.8)	0.2 (0.1–0.4)	2.5 (0.9–7.0)
Water birth	2.4 (1.2–4.6)	2.7 (1.9–3.8)	0.9 (0.4–1.9)
Upright position for birth	1.5 (0.8–3.1)	1.9 (1.4–2.7)	0.8 (0.4–1.7)

### Primary outcomes

Overall, women intending birth in an FMU were significantly more likely to have a spontaneous, uncomplicated birth with good outcome for both mother and infant, compared to women in the OU group (OR 2.6; 95% CI 2.0–3.4). Analysis by parity confirmed this effect of place of birth for both primiparous (OR 2.2; CI 1.4–3.3) and multiparous women (OR 2.9; CI 2.0–4.2). The effect for primiparous and multiparous women was not significantly different (OR 0.7; CI 0.4–1.3).

Compared with women planning to give birth in an OU, women in the FMU group were significantly less likely to undergo caesarean section (OR 0.5; CI 0.3–0.9), an effect which was confirmed for primiparous women (OR 0.4, CI 0.2–0.9). A similar trend was also found for multiparous women (OR 0.8; CI 0.3–2.2) although the difference was non-significant. The statistical analysis revealed no significant difference in effect between the parity-induced subgroups (effect ratio OR 0.6; CI 0.2–2.1).

### Secondary perinatal outcomes

There were no overall, statistically significant differences in Apgar score <9/5 min, NICU admission >24 h or infant readmission between the two groups; nor were any found in the comparison of groups by parity.

### Secondary maternal outcomes

Women in the FMU group were significantly more likely than women in the OU group to have an intact perineum after delivery (OR 1.3; CI 1.1–1.6), while no significant difference was found for third/fourth-degree tears (OR 0.8; CI 0.4–1.4). In similarity to the findings of the overall study, multiparous women in the FMU group were significantly more likely to avoid perineal injury compared to the corresponding OU group (OR 1.3; CI 1.02–1.7). While failing to reach statistical significance, the same trend was found for primiparous women (OR 1.3; CI 0.9–1.9). The effect ratio was OR 1.0; CI 0.6–1.6. For third/fourth-degree tearing, no significant effect differences were found between subgroups.

Compared to the women in the OU group, women in the FMU group were significantly less likely to be readmitted within 28 days postpartum (OR 0.6; CI 0.4–0.99). The same applied for the primiparous women (OR 0.3; CI 0.1–0.9), while a similar trend for multiparous women did not reach statistical significance (OR 0.8; CI 0.4–1.4). No significant effect difference by parity was found (effect ratio OR 0.4; CI 0.1–1.3).

### Birth interventions

A comparison of women in the FMU group with women in the OU group showed the former to have significantly fewer instrumental deliveries (OR 0.4; CI 0.2–0.6) and augmentations of labour (OR 0.4; CI 0.3–0.5). Similar significant results were obtained when comparing the two groups by parity. Instrumental deliveries were thus significantly less frequent among primiparous (OR 0.4; CI 0.2–0.7) and multiparous (OR 0.3; CI 0.1–0.9) women in the FMU group, compared to their OU counterparts. The same applied for labour augmentation: primiparas (OR 0.4; CI 0.3–0.6) and multiparas (OR 0.3; CI 0.2–0.5). Effect ratios failed to show significant differences for instrumental delivery (OR 1.5; CI 0.4–6.2) and labour augmentation (OR 1.3; CI 0.6–2.7).

### Other secondary outcomes

Epidural analgesia was used significantly less frequently in FMUs compared to OUs (OR 0.3; CI 0.2–0.5). Similar significant trends were found for primiparous (OR 0.4; CI 0.3–0.8) and multiparous (OR 0.2; CI 0.1–0.4) women (effect ratio OR 2.5; CI 0.9–7.0).

Conversely, water birth (OR 2.6; CI 1.9–3.5) and upright birth position (OR 1.9; CI 1.4–2.5) were significantly more prevalent in the FMU group than in the OU group. For water birth, the effect was confirmed for both primiparas (OR 2.4; CI 1.2–4.6) and multiparas (OR 2.7; CI 1.9–3.8). The effect ratio was OR 0.9; CI 0.4–1.9. A significantly higher incidence of upright birth position was also found among multiparous women in the FMU group (OR 1.9; CI 1.4–2.7), while a similar although non-significant trend was found for primiparous women (OR 1.5; CI 0.8–3.1). The effect ratio was OR 0.8; CI 0.4–1.7.

### Transfers

**Fig. 1:**
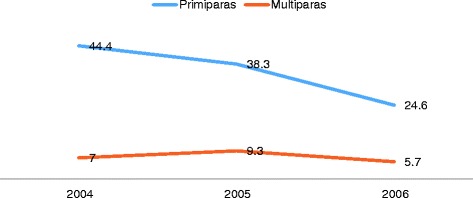
Yearly transfer rates by parity

Of the 839 women in the FMU group, 124 (14.8%) were transferred to an OU during birth or within 2 h after birth, but substantial differences in transfer rates were observed between primiparas and multiparas. Thus, 36.7% of primiparas were transferred, compared with 7.2% of multiparas. As shown in Fig. 1, primiparas’ transfer rate declined from 44.4% in 2004 to 24.6% in 2006, while little change was seen for multiparous women.

**Fig. 2:**
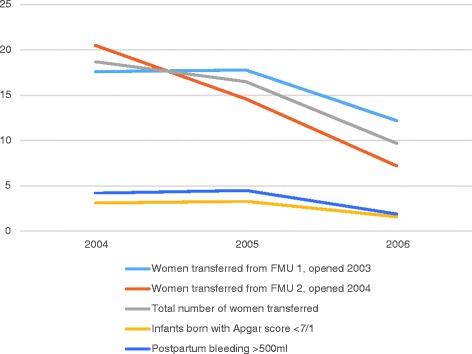
Transfer rates by FMU and rates of Apgar scores <7/1 and postpartum bleeding >500 ml

As Fig. 2 shows, the transfer rates for FMU 1, which had opened a year before the initiation of the Danish birth centre study, declined steadily during the study period, while transfers remained at a high level during the first year for FMU 2, which had opened in March 2004 (when the inclusion period began). After the first year, a sharp and steady decline was seen. In both FMUs, the study period saw slight declines in the incidence of Apgar score <7 at 1 min and postpartum bleeding >500 ml.

**Fig. 3:**
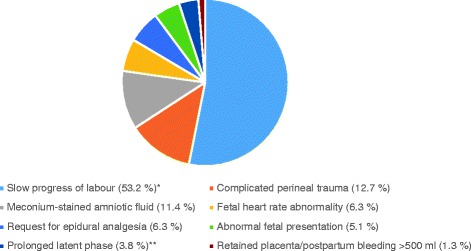
Reasons for transfer during labour or <2 h postpartum: primiparas. *Defined as no progress for >2 h in 1^st^ stage or active pushing for >2 h in 2^nd^ stage. **Defined as painful contractions >24 h and cervical dilation <3 cm

Regardless of parity, the most common reason for transfer was slow progress of labour. However, the overall transfer rate on this indication declined from 50 to 33.3% over the study period, while transfer rates for each of the other indications fluctuated slightly around a mean rate. For primiparas, slow progress in the first or second stages of labour alone accounted for more than half of transfers (53.2%; Fig. 3). For multiparas, 28.9% of transfers were carried out on this indication, the second most frequent reason being postpartum haemorrhage >500 ml (17.8%).

## Discussion

### Main findings

Low-risk primiparous women intending to give birth in an FMU rather than in an OU were found to have a significantly higher incidence of a spontaneous, uncomplicated birth with good outcomes for mother and infant and significantly lower risk of intrapartum caesarean section. These women were furthermore significantly less likely to require labour augmentation, epidural analgesia, instrumental delivery or hospital readmission, while they were more likely to have a water birth. We found no indication that parity modified the effect of birthplace on the maternal and perinatal birth outcomes under investigation. As for transfer rates, substantial differences were observed between primiparous and multiparous women (36.7 and 7.2%, respectively), with slow progress of labour being the most frequent reason, irrespective of parity.

### Strengths

An overall strength of the study is that all participating units operated under identical practice guidelines, in a publicly funded health care system with midwives as primary care providers for all low-risk births, thus minimising confounding by differences across birth settings in clinical practice, care provision and economic determinants of birthplace. Similarly, confounding due to differences in obstetric risk factors was of little concern as all the included women were assessed to be at low risk at the start of care in labour.

Moreover, a major strength of this study is the inclusion of all eligible women admitted to the participating FMUs during the study period, with no loss to follow-up, thereby providing a complete, high-quality data set.

### Limitations

The non-randomised design represents an important limitation of the study. Despite the close matching on potential confounding factors and the restriction to low-risk women, the possibility of residual confounding cannot be excluded given the observational study design.

Further, an unexpected closure of the two participating FMUs posed a major challenge to the original study, preventing the harvest of the originally intended data amount. However, post hoc recalculation revealed only a modest loss of statistical power. Additional details are described in previous publications [37, 48]. As for the subgroup analysis presented here, primiparous women constituted only 25.6% of all participants; it may thus be questioned whether the statistical power of our study is sufficient to detect true differences between subgroups. The risk is nonetheless considered low, as the confidence intervals are relatively narrow.

A further potential limitation is the age of our dataset. However, the rigorous assessment criteria used for low obstetric risk are in line with the more recent and internationally accepted NICE guidelines [55] and may thus be considered up-to-date. The potential confounders, such as maternal age and pregestational BMI, have moreover maintained a steady level among women in the region [57, 58].

### Interpretation of findings

Patient safety has been a central issue in debates over care in FMUs [32–35], with primiparas attracting special attention due to their relatively high rate of obstetric complications and transfers. Our study found no significant effect differences by parity for any of the investigated maternal and perinatal outcomes, indicating that FMUs serve primiparous and multiparous women equally well.

Freestanding midwifery units aim to offer low-risk women a choice among birth places without compromising safety for themselves or their infants. Severe complications and adverse outcomes are, however, difficult to measure in studies comparing different birth settings, as their occurrence is rare in women at low obstetric risk. The use of a strictly positive composite outcome allowed us to capture rare events. The finding that intention to give birth in an FMU rather than in an OU more than doubled their chance of having a spontaneous, uncomplicated birth with good outcome for mother and infant should be reassuring for primiparous women. Optimal positive outcomes of birth are thus entirely attainable for women attracted by alternatives to birth in a traditional OU setting.

For primiparous women planning to give birth in an FMU, the likelihood of intrapartum caesarean section was 60% lower than for primiparas planning to give birth in an OU. This result is consistent with the findings of the extensive Birthplace in England study and the research on freestanding midwifery units in Quebec [8, 44]. The result is likewise in line with the reduced use of interventions for all women generally documented by studies of care in FMUs [59]. It is increasingly being recognised that the mode of delivery in the first birth influences delivery mode and outcomes in subsequent births [19, 49–53, 60, 61]. This strongly suggests that the key to curbing the increasing use of caesarean section lies in preventing the need for first-birth caesarean deliveries [20–23, 25, 26]. This stresses the importance of our finding of a substantial reduction in caesarean section among primiparous women.

The main difference between primiparous and multiparous women was found in their respective transfer rates. Our finding of markedly higher rates for primiparas than for multiparas corroborates earlier work [36, 45, 46]. The studies cited also agree with our finding that the most frequent indication for transfer in both primiparous and multiparous women was a slow progress of labour. For safety reasons, the criteria for transfer on the indication of slow labour progress were rather strict in the participating FMUs (no progress for 2 h, see Fig. 3), which is likely to have contributed to the high transfer rate on this indication.

This study is the first to report the finding of a steep decline in overall transfer rates for primiparous women during the relatively short period of 2.5 years. As this occurred simultaneously with a decline in transfers of primiparous women on indication of slow progress of labour, we find this development likely to reflect the increasing experience gained by midwives in supporting normal birth and the positive influence of feedback from regular multidisciplinary audits with attendance of both FMU and OU staff. Another contributing reason may be that part of the FMU 2 data were collected from its opening day and thus during the units start-up phase, in which it may have been underperforming.

The decline in transfer rates was not associated with a concomitant increase in the incidence of Apgar score <7 at 1 min and postpartum haemorrhage >500 ml. In general, our results on maternal and perinatal outcomes suggest that the referral and transfer system represents an effective safety net to support both primiparous and multiparous women wishing to give birth in an FMU.

Overall, the present study adds to the limited body of evidence concerning the suitability of care in FMUs for primiparous women. Our findings may be relevant to birthing women as well as to health professionals and policy makers in the planning of maternity care services. The results of this and similar research suggest that, regardless of parity, care in an FMU is a safe alternative to care in an OU for low-risk women, and that care in FMUs offers important benefits for both primiparous and multiparous women. Furthermore, the reported reduction in caesarean delivery, both overall and in primiparous childbirth, indicates that FMUs may hold an untapped potential to halt or even reverse the global rise in the use of caesarean section.

Attempts to extend the validity of our results to other populations and regions should be regarded with circumspection. The participants in this study were drawn from an ethnically and culturally homogenous population of women with free access to all national maternity care services and; our results may therefore not be directly applicable to relatively more diverse populations. The quality and safety measures implemented in the participating units should also be taken into account. We would emphasize the importance of the training and experience of FMU midwives in managing obstetric emergencies, the high standard of transfer guidelines and the regular audits between FMU and OU staff on the quality of care.

## Conclusions

Irrespective of parity, the intention to give birth in an FMU rather than in an OU significantly raised the likelihood of having a spontaneous, uncomplicated birth with good outcome for mother and infant. No effect differences by parity were found for any outcome. For primiparas, the likelihood of intrapartum caesarean section was less than half in FMUs than in OUs. Transfer rates for multiparas were moderate and stable during the study period, while primiparas were transferred far more often although numbers declined substantially over the study period.

Our results indicate that care in FMUs offers advantages over care in OUs to both primiparous and multiparous mothers, while their infants are equally safe in both settings. The provision of care in FMUs as an alternative to care in OUs for all low-risk women, regardless of parity, thus finds support here.

## Additional file

Additional file 1: Study flow chart. (DOCX 80 kb)

## Abbreviations

BMI: Body mass index
FMU: Freestanding midwifery unit
NICE: National Institute for Health and Care Excellence
NICU: Neonatal intensive care unit
OU: Obstetric unit
