# Supplementary figures and images for: Are freestanding midwifery units a safe alternative to obstetric units for low-risk, primiparous childbirth? An analysis of effect differences by parity in a matched cohort study
Source: BMC Pregnancy Childbirth. 2017 Jan 9;17:14. doi: 10.1186/s12884-016-1208-1 (PMC5223304; doi:10.1186/s12884-016-1208-1)

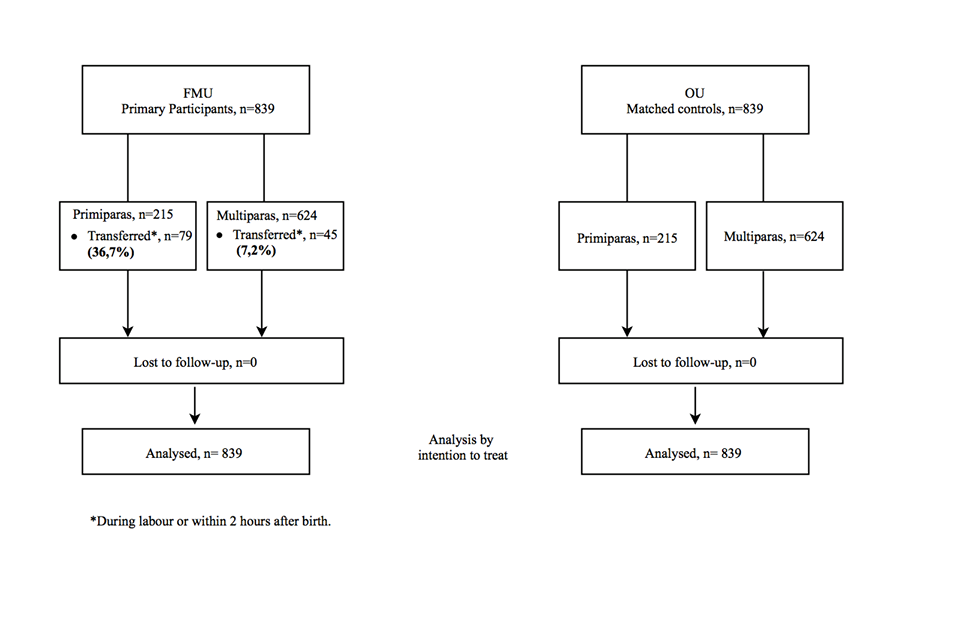

Supplement: Additional file 1: — Study flow chart. (DOCX 80 kb) [file 12884_2016_1208_MOESM1_ESM.docx]
